# Oral conditions are associated with salt taste disability among American adults

**DOI:** 10.1111/odi.14926

**Published:** 2024-04-02

**Authors:** Luisa Schertel Cassiano, Fábio R. M. Leite, Silas Alves‐Costa, Susilena Arouche Costa, Gustavo G. Nascimento

**Affiliations:** ^1^ Department of Dentistry and Oral Health Aarhus University Aarhus Denmark; ^2^ National Dental Research Institute Singapore National Dental Centre Singapore Singapore Singapore; ^3^ Oral Health Academic Clinical Programme Duke‐NUS Medical School Singapore Singapore; ^4^ Graduate Program in Dentistry Federal University of Maranhão São Luís Brazil

**Keywords:** chemosensory disorders, dental caries, edentulism, periodontal diseases, taste, xerostomia

## Abstract

**Aim:**

To explore the association between oral conditions and their interaction with salt taste disability among American adults.

**Methods:**

Data from the 2013–2014 NHANES cycle were used (*n* = 2373). The exposures were periodontitis, defined by the 2017 EFP‐AAP classification, dental caries, missing teeth, and edentulism, as per the DMF‐T index, and xerostomia. The outcome was salt taste disability, objectively assessed. Covariates included sex, age, educational level, poverty index, obesity, diabetes, smoking, alcohol consumption, and medications related to mouth dryness. Weighted multivariable logistic regression modeling was used to evaluate the relationship between oral conditions and their interaction and salt taste disability.

**Results:**

Participants who reported xerostomia were more likely to have salt taste disability (OR 2.42; 95%CI 1.44–4.07), especially those older than 60 years (OR 3.63; 95%CI 1.72–7.63). Among participants aged 40–59, xerostomia increased the chance of salt taste disability; however, the confidence interval included the null value. The interactions between xerostomia and edentulism increased the chance of salt taste disability.

**Conclusion:**

Oral conditions seem to influence the ability to taste salt. Dental professionals may help identify individuals with taste alterations and raise their awareness of the risk of systemic diseases that require the reduction of salt intake.

## 1 INTRODUCTION

Taste perception refers to how individuals perceive and discriminate chemical stimuli like flavors (Dobrow et al., 2021). In the United States, distorted taste perception can be found in 17% of the adult population and is more common among older persons (Liu et al., 2016). Taste impairment has been associated with an increased mortality risk, as it impacts one's ability to detect environmental hazards (Reed & Knaapila, 2010), such as rotten food.

Taste perception also influences diet and food intake (Boesveldt & de Graaf, 2017), which, in turn, has been associated with the development of non‐communicable diseases (Catamo et al., 2021). For instance, hypertension, a major cause of cardiovascular disease and overall mortality (Lewington et al., 2002), may result from high dietary sodium intake (He et al., 2013), which appears to be determined by the perception of salt taste. Studies have shown that impaired salt taste ability was associated with higher sodium intake and urine excretion after 24 h (Piovesana et al., 2013). Additionally, distorted salt taste perception has been related to a higher prevalence of high blood pressure, as evidenced by increased systolic and diastolic blood pressure levels among Asian adults (Cho et al., 2016). Using data from two Japanese communities, Kudo and coworkers found that individuals with a decreased ability to detect salt taste had higher salt intake and blood pressure levels (Kudo et al., 2021). Although there appears to be a genetic predisposition to salt sensitivity, the response to salt taste results from an intricate interplay of several factors (Beauchamp & Engelman, 1991). While aging and some systemic diseases, like diabetes (Catamo et al., 2021) and hypertension (Liu et al., 2018), relate to a distorted sense of taste, whether oral conditions are associated with taste disability remains to be explored (Batisse et al., 2017).

A negative impact on taste ability has been associated with various oral conditions. These include trauma due to anesthesia or oral surgery (Shintani et al., 2020), inflammation‐related disorders including burning mouth (Su et al., 2020), oral lichen planus (Suter et al., 2017), benign migratory glossitis (Ching et al., 2012), and infectious oral diseases like candidiasis (Sakashita et al., 2004) and viral infections (Risso et al., 2020). Periodontitis, a destructive inflammatory disease that affects the supporting structures of the teeth, and halitosis, a clinical sign of periodontitis, have been associated with taste impairment (Schertel Cassiano, Leite, et al., 2023; Schertel Cassiano, Ribeiro, et al., 2023). Moreover, xerostomia, the subjective feeling of a dry mouth, has been related to impaired taste (Hummelsheim et al., 2021; Kamel et al., 2009). However, there is no evidence on whether edentulism, tooth loss, and dental caries are associated with taste perception. Previous studies have hypothesized a biological model to explain the relationship between oral conditions and distorted taste, comprising inflammation‐related factors, and physical paths, such as abundant tongue coating acting as a barrier for perceiving taste molecules and lack of saliva to transport and dissolve taste substances (Matsuo, 2000; Schertel Cassiano, Leite, et al., 2023). On that note, one may speculate whether tooth conditions may also play a part in this relationship, as tooth absence may lead to chewing disability because individuals are not able to properly break down the food into small particles that can stimulate the taste receptors.

While studies exploring the part played by some oral conditions, mainly xerostomia, in the occurrence of taste impairment may exist, to date, there are no investigations on the interaction between these oral conditions, which are likely to co‐occur especially among older adults (Lamster et al., 2016). In addition, most studies on the topic have focused on specific samples (e.g., Sjögren's syndrome or under chemo‐ or radiotherapy), and information from the general population is scant. Accordingly, we aimed to investigate the association between periodontitis, dental caries, tooth loss, edentulism, and xerostomia with salt taste disability among American adults. We hypothesize that oral conditions and their interaction will be associated with salt taste disability.

## 2 METHODS

### 2.1 Study design and participants

We analyzed data from the National Health and Nutrition Examination Survey (NHANES) 2013–2014 cycle. NHANES is a cross‐sectional population‐based study that uses complex, multi‐stage, stratified representative sampling of non‐institutionalized American civilians. A sample weight was attributed to each sample person, comprising a measure of the number of persons in the population represented by that sample person in NHANES. It reflects the unequal probability of selection, adjustment for sample person non‐response, and an adjustment to account for differences between the final sample and the total population based on independent population control totals. This NHANES cycle was chosen because it is the most extensive dataset, including oral and taste exams. Adults over 40 years old who have completed dental and taste perception examinations were eligible for this study (*n* = 2373). Participants who could not provide a correct ordinal ranking of the three light intensity standards on the general Labelled Magnitude Scale (gLMS), reported some health condition requiring antibiotic prophylaxis before periodontal probing, or who were pregnant or breastfeeding at the time of the assessment were excluded.

### 2.2 Exposures—Oral conditions

Prior to dental examinations, dental examiners underwent comprehensive training and calibration, including periodic monitoring and recalibration, to ensure quality oral health data. Details regarding the training and calibration process are available elsewhere (Dye et al., 2019). For dental caries in permanent teeth, Kappa scores were 0.93 and 0.96 during 2011–2014. For untreated dental caries, the Kappa scores were 0.82 and 0.91 during the same period. The Kappa scores for 4 and 6 mm thresholds of clinical attachment loss (CAL) ranged from 0.52 to 0.66 during 2011–2014. For overall CAL and periodontal probing depth (PPD) across all periodontal sites, the inter‐class coefficients (ICCs) ranged from 0.80 to 0.90 and from 0.79 to 0.86, respectively, during 2011–2012 and 2013–2014.

Full‐mouth periodontal examination was carried out if at least one natural permanent tooth was present. Gingival recession and PPD measures were performed at six sites per tooth (excluding third molars) using a Hu‐Friedy (Chicago, IL, USA) periodontal probe. An algorithm calculated the CAL using those measurements. All four quadrants were examined, and all measurements were rounded to the lowest whole millimeter. In each cycle, the periodontal assessment was performed by calibrated dentists (Dye et al., 2019). Periodontitis was classified according to the European Federation of Periodontology and the American Academy of Periodontology (EFP‐AAP), proposed at the World Workshop on the Classification of Periodontal and Peri‐implant Diseases and Conditions in 2017 (Papapanou et al., 2018). For analytical purposes, this condition was further dichotomized into healthy (reference category) versus participants with stage II, III, and IV periodontitis.

Each participant aged 1 year and older received the coronal caries assessment. All teeth except the third molars were assessed. Each quadrant was dried with air and examined with a surface‐reflecting mirror and a No. 23 explorer. Diagnostic criteria for the coronal caries examinations were those developed in the Proceedings of the Conference on Clinical Testing of Cariostatic Agents, sponsored by the American Dental Association in 1968. The D component of the DMFT index was used to calculate the number of decayed teeth. Individuals were categorized as 0—no decayed teeth and 1—the presence of at least one decayed tooth. The M component was used to record the number of missing teeth. NHANES uses three codes to distinguish the reason for missing teeth: code “E”: teeth extracted due to caries and periodontitis; code “M”: teeth extracted due to trauma, orthodontic treatment, or other non‐disease related causes; code “U”: unerupted or congenitally missing teeth. For this study, only missing teeth related to the code “E” (disease‐related) were considered. Thus, the participants were classified as having no missing teeth due to oral diseases; and at least one missing tooth due to oral diseases. In addition, individuals with >20 missing teeth were also identified (as this measure is also relevant for periodontal purposes—Stage IV). Those individuals completely edentulous were compared to those not completely edentulous participants.

Xerostomia was identified upon self‐reporting of persistent dry mouth during the last year (no/yes).

### 2.3 Outcome—Salt taste disability

Objectively measured salt taste ability was assessed after participants were asked to take 10 mL of the 0.32 M NaCl solution in their mouths, gently swish the solution for 3 seconds, and then spit it out without swallowing it. Individuals who failed to correctly identify the salty taste (bitter/something else/no taste/sour) were considered to have salt taste disability.

### 2.4 Covariates

Questionnaires were used in each NHANES cycle to collate information on sex (male/female), age (years), and educational level (<9th grade/9–11th grade and 12th grade with no diploma/high school graduate or GED or equivalent/some college or AA degree/ college graduate or above). In addition, the family income to poverty ratio (FIPR), ranging from 1 to 5, based on the federal poverty threshold established by the US Census Bureau in the respective year, was categorized into low income 1.3, middle income (>1.3 to ≤3.5), and high income (<3.5). Smoke exposure was identified when people reported smoking at least 100 cigarettes (yes/no).

Stadiometer and digital weight scale were used to estimate the body mass index (BMI), which was classified as underweight (<18.5 kg/m^2^), normal weight (≥18.5–24.9 kg/m^2^), overweight (25–29.9 kg/m^2^), and obese (≥30 kg/m^2^). Diabetes status was identified according to the percentage of glycosylated hemoglobin as normal (≤5.6%), prediabetic (5.7%–6.4%), and diabetic (≥6.5%).

Medications related to mouth dryness were obtained and included antihistamines, decongestants, antidepressants, antipsychotics, antihypertensives, and anticholinergics (Shetty et al., 2012). Individuals were categorized as using at least one medication related to mouth dryness. Finally, alcohol consumption was also included in the analysis (>4 drinks/occasion for women and >5 drinks/occasion for men in the past 30 days) (Wilsnack et al., 2018).

### 2.5 Analytical approach

**FIGURE 1:**
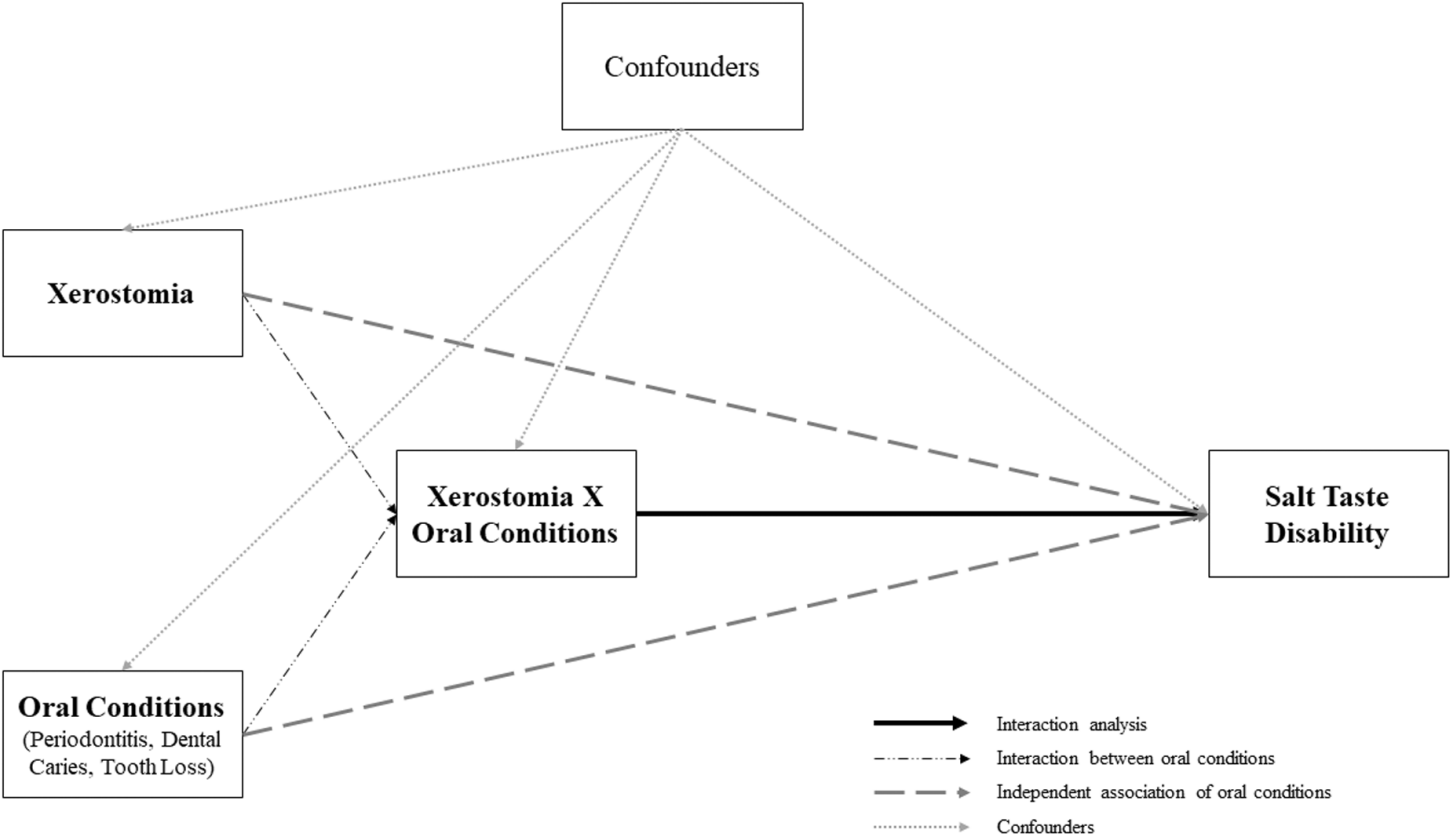
Directed acyclic graph depicting the association between oral conditions and their interaction and salt taste disability among American adults. Confounders include sex, age, educational level, FIPR, BMI, smoking, diabetes, alcohol consumption, and medications related to mouth dryness.

The analytical approach was guided by the directed acyclic graph (DAG) displayed in Figure 1. We assumed that xerostomia and the other oral conditions function as independent exposures of the outcome, salt taste disability. In addition, we also tested their interaction with xerostomia. The above‐described covariates were included in the analytical model as confounders of the association between oral conditions and salt taste disability, as no covariate was located in the presumed causal path between exposure and outcome, thus reducing the likelihood of potential colliders and back‐door paths. The detailed DAG depicting the relationship between all variables is given in Figure S1.

Weighted proportions with their 95% confidence intervals (CIs) were analyzed according to the oral status using the chi‐squared test for categorical variables and the t‐student test for age (in years). Additionally, weighted multiple multivariable logistic regression models were used to evaluate the relationship between periodontitis, dental caries, tooth loss, edentulism, and xerostomia and their interaction (by adding an interaction term between the conditions and xerostomia in the analytical models) with salt taste disability (Figure 1). Adjusted odds ratios (ORs) and their respective 95%CIs adjusted for the covariates were estimated. In addition, analyses were stratified by age (40–59 years and ≥60 years).

A sensitivity analysis for unmeasured confounding was performed based on the *E*‐value calculation (VanderWeele & Ding, 2017). A large *E*‐value implies that considerable unmeasured confounding would be required to eliminate the association between the exposure and outcome. Analyses were carried out using Stata 16.1 using the “*svy*” command for complex survey analyses.

## 3 RESULTS

Table 1 displays the proportion with its respective standard error (SE) of the explanatory variables according to the oral conditions. On average, participants with periodontitis were 56.5 (SE 0.5) y‐old, and with xerostomia, 60.6 (SE 0.8) y‐old. Salt taste disability was more prevalent among participants with xerostomia, dental caries, missing teeth, and edentulism.

**TABLE 1:** The weighted characteristics proportion of Americans according to the oral status, NHANES 2013–2014 (*n* = 2373).

	Xerostomia		Periodontitis		Caries		Missing teeth		>20 missing teeth		Edentulous	
	Proportion (SE)		Proportion (SE)		Proportion (SE)		Proportion (SE)		Proportion (SE)		Proportion (SE)	
	Yes	No	Yes	No	Yes	No	Yes	No	Yes	No	Yes	No
Sex												
Male	9.0 (1.1)	91.0 (1.1)	30.0 (2.7)	70.0 (2.7)	23.3 (2.1)	76.7 (2.1)	65.3 (2.3)	34.7 (3.3)	5.3 (0.7)	44.3 (0.8)	7.4 (1.2)	92.6 (1.2)
Female	16.3 (1.4)	83.7 (1.4)	20.3 (2.6)	79.7 (2.6)	20.4 (2.1)	79.6 (2.1)	64.4 (2.6)	35.6 (2.6)	5.8 (1.0)	44.5 (1.4)	8.2 (1.7)	91.8 (1.7)
Age, years^a^, mean (SE)	60.6 (0.8)	56.3 (0.3)	56.5 (0.5)	57.0 (0.4)	54.2 (0.5)	57.6 (0.4)	59.7 (0.3)	51.8 (0.4)	65.2 (0.5)	55.8 (0.2)	65.7 (0.7)	56.2 (0.3)
Educational level												
<9th grade	19.4 (3.8)	80.6 (3.8)	38.1 (6.7)	61.9 (6.7)	35 (5.5)	65.0 (5.5)	85.4 (4.9)	14.6 (4.9)	0.8 (0.1)	2.7 (0.5)	14.7 (2.3)	85.3 (2.3)
9–11th and 12th grade with no diploma	20.9 (2.4)	79. 1 (2.4)	36.6 (4.3)	63.4 (4.3)	39.9 (4.1)	60.1 (4.1)	87.0 (2.1)	13.0 (2.1)	2.7 (0.6)	7.0 (0.8)	22.2 (3.9)	77.8 (3.9)
High school graduate or GED or equivalent	15.1 (2.1)	84.9 (2.1)	30.8 (4.3)	69.2 (4.3)	30.3 (3.9)	69.7 (3.9)	80.1 (2.6)	19.9 (2.6)	3.5 (0.6)	18.4 (1.2)	11.4 (1.9)	88.6 (1.9)
Some college or AA degree	13.9 (1.8)	86.1 (1.8)	25.3 (2.7)	74.7 (2.7)	21.4 (1.8)	78.6 (1.8)	67.8 (2.3)	32.2 (2.3)	3.3 (0.4)	27.6 (1.6)	6.8 (1.2)	93.2 (1.2)
College graduate or above	6.8 (1.0)	93.2 (1.0)	16.5 (2.3)	83.5 (2.3)	10.0 (1.7)	90.0 (1.7)	43.6 (3.4)	56.4 (3.4)	0.7 (0.2)	32.9 (2.6)	1.4 (0.6)	98.6 (0.6)
Family income to poverty ratio												
Low income	23.2 (2.0)	76.8 (2.0)	34.7 (4.1)	65.3 (4.1)	39.0 (3.2)	61.0 (3.2)	80.8 (3.1)	19.2 (3.1)	4.3 (0.8)	13.9 (1.9)	15.7 (1.9)	84.3 (1.9)
Middle income	14.3 (1.3)	85.7 (1.3)	30.3 (3.0)	69.7 (3.0)	28.1 (2.4)	71.9 (2.4)	77.2 (1.8)	22.8 (1.8)	4.2 (0.5)	29.5 (1.1)	8.8 (1.4)	91.2 (1.4)
High income	7.5 (1.0)	92.5 (1.0)	17.9 (2.3)	82.1 (2.3)	10.8 (1.8)	98.2 (1.8)	50.1 (3.5)	49.9 (3.5)	2.5 (0.6)	45.4 (3.6)	4.1 (1.0)	95.9 (1.0)
BMI												
Underweight	30.1 (12.4)	69.9 (12.4)	25.4 (11.5)	74.6 (11.5)	30.6 (11.6)	69.4 (11.6)	30.6 (11.6)	69.4 (11.6)	0.5 (0.1)	0.5 (0.1)	36.2 (12.5)	63.8 (12.5)
Normal weight	11.0 (1.9)	89.0 (1.9)	21.4 (3.2)	78.6 (3.2)	18.3 (2.5)	81.7 (2.5)	18.3 (2.5)	81.7 (2.5)	2.4 (0.4)	21.7 (0.9)	7.3 (1.6)	92.7 (1.6)
Overweight	10.8 (1.4)	98.2 (1.4)	22.2 (2.4)	77.8 (2.4)	17.1 (1.9)	82.9 (1.9)	17.1 (1.9)	82.9 (1.9)	3.6 (0.4)	31.9 (1.5)	7.2 (1.4)	92.8 (1.4)
Obese	14.9 (1.5)	85.1 (1.5)	29.8 (3.3)	70.2 (3.3)	27.9 (2.4)	72.1 (2.4)	27.9 (2.4)	72.1 (2.4)	4.5 (0.7)	34.5 (1.9)	7.9 (1.7)	92.1 (1.7)
Smoking												
No	10.1 (1.3)	89.9 (1.3)	21.4 (2.7)	78.6 (2.7)	16.3 (2.0)	83.7 (2.0)	54.6 (3.4)	45.4 (3.4)	6.6 (0.8)	74.5 (2.4)	3.7 (0.1)	96.3 (0.1)
Yes	15.4 (0.9)	84.6 (0.9)	29.2 (2.9)	70.8 (2.9)	27.9 (2.1)	72.1 (2.1)	76.2 (2.1)	23.8 (2.1)	4.4 (0.8)	14.3 (1.2)	12.3 (1.8)	87.7 (1.8)
Diabetes												
Normal	10.3 (0.7)	89.7 (0.7)	24.4 (2.6)	75.6 (2.6)	20.8 (1.8)	79.2 (1.8)	62.1 (2.9)	37.9 (2.9)	7.7 (1.1)	71.8 (1.8)	7.1 (1.3)	92.9 (1.3)
Prediabetic	12.9 (3.2)	87.1 (3.2)	23.8 (4.2)	76.2 (4.2)	20.1 (4.6)	79.9 (4.6)	64.8 (5.8)	35.2 (5.8)	0.7 (0.1)	5.6 (0.7)	6.5 (1.4)	93.5 (1.4)
Diabetic	26.0 (3.5)	74.0 (3.5)	29.7 (4.1)	70.3 (4.1)	28.3 (3.0)	71.7 (3.0)	80.7 (2.1)	19.3 (2.1)	2.6 (0.5)	11.4 (0.8)	12.2 (3.0)	87.8 (3.0)
Alcohol												
No	6.7 (0.4)	56.3 (3.0)	20.7 (1.0)	42.4 (2.0)	10.8 (1.2)	52.2 (2.7)	37.3 (2.2)	25.8 (2.4)	4.4 (0.7)	58.7 (3.6)	3.2 (0.6)	59.9 (3.5)
Yes	5.8 (0.7)	30.9 (2.6)	15.2 (1.0)	21.5 (2.0)	10.9 (0.9)	25.9 (2.6)	27.5 (2.4)	9.3 (1.0)	6.6 (0.9)	30.1 (2.5)	4.5 (0.7)	32.2 (2.6)
Medications												
No	10.9 (0.6)	70.4 (1.1)	28.1 (1.4)	53.3 (1.5)	17.5 (1.0)	63.8 (1.0)	53.4 (2.2)	27.9 (2.2)	9.6 (1.4)	71.7 (1.7)	6.9 (1.2)	74.5 (1.6)
Yes	1.7 (0.3)	16.8 (0.7)	7.8 (0.09)	10.7 (0.7)	4.2 (6.0)	14.3 (8.0)	11.3 (0.8)	7.2 (0.5)	1.4 (0.3)	17.1 (0.7)	0.8 (0.1)	17.7 (0.6)
Salt taste disability												
No	11.7 (0.8)	88.3 (0.8)	25.4 (2.6)	74.6 (2.6)	21.7 (1.9)	78.3 (1.9)	64.8 (2.4)	35.2 (2.4)	10.4 (1.5)	84.7 (1.6)	7.4 (1.1)	92.6 (1.1)
Yes	19.9 (4.1)	80.1 (4.1)	23.1 (3.2)	76.9 (3.2)	22.9 (2.9)	77.1 (2.9)	65.5 (6.7)	34.5 (6.7)	0.6 (0.1)	4.1 (0.6)	10.5 (3.9)	89.5 (3.9)

Abbreviations: BMI, body mass index; SE, standardized error.

^a^ Means distribution, continuous variable.

Table 2 describes the adjusted odds ratios (OR) and their 95% confidence intervals (CI) of signs of salt taste disability from weighted multiple multivariable logistic regression analyses. The chance of having salt taste disability increased among participants who reported xerostomia (OR 2.42; 95%CI 1.44–4.07). Additionally, the interactions between xerostomia and periodontitis, xerostomia and caries, and xerostomia and edentulism were noted, which increased the chance of salt taste disability upon their cooccurrence. Participants experiencing edentulism and xerostomia had the highest odds ratio for salt taste disability (OR 4.03; 95%CI 1.18–13.77).

**TABLE 2:** Adjusted odds ratios^a^ of oral conditions and salt taste disability among Americans, NHANES (2011–2014).

	Salt taste disability	
	OR (95%CI)	*E*‐value (lower CI limit)
Periodontitis	0.87 (0.57–1.33)	–
Dental caries	1.29 (0.71–2.34)	–
Missing teeth	0.99 (0.52–1.89)	–
>20 missing teeth	0.96 (0.44–2.07)	–
Edentulism	0.86 (0.39–1.90)	–
Xerostomia	**2.42 (1.44–4.07)**	3.39 (1.49)
Periodontitis # Xerostomia	**2.17 (1.03–4.56)**	4.09 (1.74)
Caries # Xerostomia	**2.16 (1.09–4.30)**	3.74 (1.40)
Missing teeth # Xerostomia	2.25 (0.79–6.40)	–
>20 missing teeth# xerostomia	1.23 (0.24–6.21)	
Edentulism # Xerostomia	**4.03 (1.18–13.77)**	7.52 (1.64)

*Note*: # Interaction term; bold coefficients mean statistical significance.

^a^ Odds ratios and their 95% confidence intervals derived from multiple multivariable logistic regression analyses adjusted for sex, age, educational level, FIPR, BMI, smoking, diabetes, alcohol consumption, and medications related to mouth dryness.

Stratified analyses by age revealed that xerostomia was associated with salt taste disability among those 60 years and older (OR 3.63; 95%CI 1.72–7.63). In individuals aged 40–59 years, the presence of xerostomia increased the likelihood of salt taste disability by 43% (OR 1.43; 95%CI 0.95–2.16), even though the confidence interval included the null estimate (Table 3). While interactions stratified by age were tested, the small sample size of each age group precluded the estimation of stable and precise models characterized by wide confidence intervals. Formal measures of model fit, however, could not be performed given the complex sample design.

**TABLE 3:** Adjusted odds ratios^a^ of oral conditions and salt taste disability among Americans, NHANES (2011–2014) stratified by age.

	Salt taste disability	
	40–59 y‐old (*N* = 1343)	≥60 y‐old (*N* = 1030)
	OR (95%CI)	OR (95%CI)
Periodontitis	1.31 (0.79–2.15)	1.27 (0.61–2.63)
Dental caries	1.10 (0.66–1.83)	1.23 (0.60–2.52)
Missing teeth	1.24 (0.73–2.08)	0.88 (0.36–2.11)
>20missing teeth	1.43 (0.67–3.06)	1.22 (0.45–3.25)
Edentulism	1.80 (0.34–9.38)	1.05 (0.41–2.65)
Xerostomia	1.43 (0.95–2.16)	**3.63 (1.72–7.63)**

*Note*: # Interaction term; bold coefficients mean statistical significance.

^a^ Odds ratios and their 95% confidence intervals derived from multiple multivariable logistic regression analyses adjusted for sex, age, educational level, FIPR, BMI, smoking, diabetes, alcohol consumption, and medications related to mouth dryness.

The sensitivity analysis for unmeasured confounding revealed that at least a 3‐fold association between an unmeasured confounder and the exposures and outcome would be necessary to eliminate the observed associations (Table 2).

## 4 DISCUSSION

Our findings suggest an association between oral conditions and salt taste disability among American adults, especially those older than 60. Our hypothesis was partially accepted, as most oral conditions alone were not associated with distorted salt taste ability, except xerostomia, which appeared to worsen taste perception. Additionally, the cooccurrence of xerostomia and the most prevalent oral conditions, including periodontitis and dental caries, did not increase the isolated effect of xerostomia. However, an interaction between xerostomia and edentulism was noticed. The uniqueness of our findings lies in exploring this relationship among American adults and may indicate associated factors with taste impairment.

Our findings revealed that xerostomia is associated with salt taste disability. While this finding is not totally novel, previous studies have relied either on small samples (Hummelsheim et al., 2021) or specific populations, such as individuals with Sjögren's syndrome (Kamel et al., 2009) or under head and neck cancer therapy (Epstein et al., 2020). In all cases, reduced saliva seems to be the underlying reason to explain these previous findings. Saliva plays a pivotal part in taste sensitivity, as it not only dissolves but also diffuses taste substances to taste receptor sites (Matsuo, 2000). In addition, salivary components, such as sodium, seem to continuously stimulate taste buds, which may increase the salt taste sensitivity threshold slightly above the salivary sodium concentrations (Delwiche & O'Mahony, 1996; Matsuo, 2000). On a related note, studies conducted among individuals with Sjögren's syndrome have found the sweet taste to be the least affected, as sweet taste sensitivity is independent of saliva, unlike salt taste (Kamel et al., 2009).

Even though periodontitis, dental caries, and tooth loss were not associated with the outcome, the interaction between edentulism and xerostomia increased the magnitude of the xerostomia association measure on a scale other than additive. Edentulism and xerostomia usually coexist, especially among older adults and vulnerable individuals, like those affected by polypharmacy and other non‐communicable diseases (Hummelsheim et al., 2021). From a biological perspective, it is possible to hypothesize some mechanisms through which this interaction acts. Edentulism prevents food from being broken down and tastants from being carried to the taste buds (Batisse et al., 2017). On that note, a study investigating taste perception among older adults demonstrated significantly lower results of salt taste perception in the group with a higher number of missing teeth, in line with our results (Alia et al., 2021). Unexpectedly, we did not observe an interaction between missing teeth and xerostomia in either age group in our study. Therefore, it is reasonable to speculate that the variable “missing teeth” used in this study, which includes individuals with one or more missing teeth, might have diluted the association of severe tooth loss, as in the case of edentulism. This is because the absence of just one or a few teeth may not significantly affect an individual's ability to chew and break down food.

Some limitations merit attention. Firstly, we included only individuals who have completed both oral and taste examinations, thus reducing the sample size. One may speculate whether the null association between most oral conditions separately with salt taste ability could be due to our sample size. While this possibility cannot be ruled out, it is very unlikely that a larger sample would change the magnitude of the association. At most, we would expect even narrower confidence intervals, which would still be far from reaching statistical significance, with the exception of xerostomia among individuals aged 40–59 years. For this group specifically, bearing in mind the Gaussian distribution of the confidence interval, an association between xerostomia and salt taste disability could be assumed, even with the inclusion of the null value in the confidence interval.

Additionally, one of our exposures, xerostomia, relied on self‐assessment, which may suffer from various biases, including, but not restricted to, recall bias. Given the broad age range of our study, older individuals, more likely to suffer from taste impairment, were also those with a higher likelihood of suffering from recall bias. Although our analyses have been adjusted for age, it is not possible to eliminate the possibility of bias. While a sensitivity analysis including only young individuals would be desirable, the small sample size undermined further analyses at the expense of unprecise results when interactions were tested. Moreover, the cross‐sectional design of our study precludes any causal assumption. Furthermore, our study did not evaluate other factors related to salt taste ability, like mental health and diet. Higher stress and cortisol levels were associated with a reduced ability to perceive the intensity of salty solutions (Ferraris et al., 2023), and taste was linked to dietary micronutrient composition (Abeywickrema et al., 2023). Whether oral conditions could contribute to these factors is yet to be explored. Finally, reverse causation is unlikely to have impacted our results, as the ability to perceive salt taste would not lead to unfavorable oral outcomes.

The strengths of this study are also worth being pointed out. NHANES uses a population‐based representative sample of the American population and standardized methods for data collection. In addition, periodontitis, dental caries, tooth presence, and taste perception could be clinically examined using well‐established methods. Furthermore, we were able to test the interaction between oral conditions and xerostomia in the general population, whereas most studies on this topic explored this relationship among specific groups. Besides, our sensitivity analysis for unmeasured confounding reinforced the robustness of our findings, as an unmeasured confounder had to be associated with both exposure and outcome on a 3‐fold scale, which seems unlikely to occur given the current set of confounders already accounted for in our study.

Further studies with a longitudinal design are still necessary to clarify biological mechanisms on this matter, and clinical trials may investigate whether therapies targeting xerostomia and other oral conditions can reverse the taste disability. Bearing in mind our current limitations, this study provides novel information that may have clinical implications. Impaired salt taste ability has been associated with a higher salt intake, which is subsequently linked to higher blood pressure (Kudo et al., 2021; Lee et al., 2014). As oral conditions seem to influence salt taste ability, dental professionals have an important role in diagnosing xerostomia and its co‐occurrence with other oral diseases. In the presence of taste alterations, individuals and respective health professionals should be informed, aiming at an interdisciplinary approach toward the management of taste disability, as well as an increased awareness of the risk of systemic conditions that require the reduction of salt intake, such as hypertension.

## AUTHOR CONTRIBUTIONS

**Luisa Schertel Cassiano:** Writing – original draft; conceptualization. **Fábio R. M. Leite:** Conceptualization; writing – review and editing; supervision. **Silas Alves‐Costa:** Writing – review and editing; formal analysis. **Susilena Arouche Costa:** Formal analysis; data curation; writing – review and editing. **Gustavo G. Nascimento:** Conceptualization; writing – review and editing; supervision; writing – original draft.

## FUNDING INFORMATION

Luisa Schertel Cassiano holds a scholarship financed by the Aarhus University Research Foundation (AUFF‐E grant# 2019‐7‐3). Silas Alves Costa and Susilena Arouche Costa received a Ph.D. scholarship from the Coordination for the Improvement of Higher Education Personnel‐Brazil (CAPES, Brazil) and a Sandwich Ph.D. scholarship from the PROCAD Amazônia‐CAPES, Brazil, Finance Code 001.

## CONFLICT OF INTEREST STATEMENT

All authors have no conflicts of interest to disclose.

## Supporting information


**FIGURE S1.** Detailed DAG depicting the relationship between oral conditions and salt taste disability and potential confounders.

## Data Availability

The data that support the findings of this study are available in NHANES at https://wwwn.cdc.gov/nchs/nhanes/continuousnhanes/default.aspx?BeginYear=2013, reference number 2013–2014. These data were derived from the following resources available in the public domain: NHANES, https://www.cdc.gov/nchs/nhanes/index.htm.
