# Supplementary figures and images for: Oral conditions are associated with salt taste disability among American adults
Source: Oral Dis. 2024 Apr 2;30(8):5475–82. doi: 10.1111/odi.14926 (PMC11610664; doi:10.1111/odi.14926)

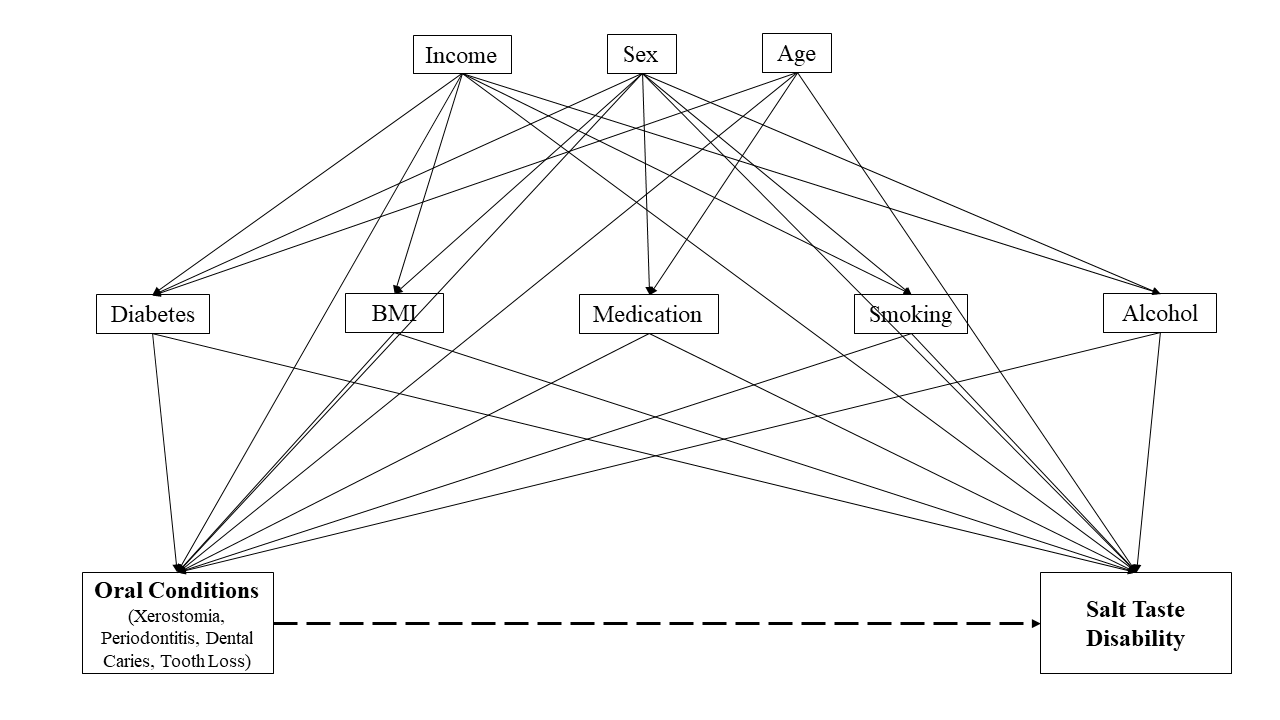

Supplement: Supplementary file 1 — FIGURE S1. Detailed DAG depicting the relationship between oral conditions and salt taste disability and potential confounders. [file ODI-30-5475-s001.tif]
